# Phase I study of neoadjuvant chemoradiotherapy with S-1 plus biweekly cisplatin for advanced gastric cancer patients with lymph node metastasis: -KOGC04-

**DOI:** 10.1186/1748-717X-9-9

**Published:** 2014-01-08

**Authors:** Satoru Matsuda, Tsunehiro Takahashi, Junichi Fukada, Kazumasa Fukuda, Hirofumi Kawakubo, Yoshiro Saikawa, Osamu Kawaguchi, Hiroya Takeuchi, Naoyuki Shigematsu, Yuko Kitagawa

**Affiliations:** 1 Department of Surgery, Keio University School of Medicine, 35 Shinanomachi, Shinjuku-ku, Tokyo 160-8582, Japan; 2 Department of Radiology, Keio University School of Medicine, 35 Shinanomachi, Shinjuku-ku, Tokyo 160-8582, Japan

**Keywords:** Gastric cancer, Neoadjuvant, Chemoradiotherapy, S-1, Cisplatin, Phase I

## Abstract

**Background:**

In patients with highly advanced gastric cancer, the recurrence rate remains high and the prognosis disappointing. We previously reported a phase I study of a neoadjuvant chemoradiotherapy of S-1 plus weekly cisplatin. Although adequate safety and efficacy were reported, myelosuppression was frequently observed, leading to treatment delay in several cases. To decrease toxicity and improve efficacy, we planned a phase I study with a modified chemotherapy regimen with biweekly cisplatin.

**Methods:**

Patients with advanced gastric cancer and lymph node metastasis who were treated by our institution between 2011 and 2012 were eligible for inclusion. The initial chemoradiotherapy schedule consisted of 6 weeks of S-1 orally administered on days 1–15 with an escalating dose of cisplatin administered on days 1 and 15. The starting dose (level 1) of cisplatin was 15 mg/m^2^, the second dose (level 2) was 20 mg/m^2^, and the third dose (level 3) was 25 mg/m^2^. Radiation of 40 Gy was administered in 20 fractions. After initial chemoradiotherapy, one cycle of combination chemotherapy with S-1 plus cisplatin was delivered. The second cycle was 42 days in duration and included S-1 administered on days 1–29 plus biweekly cisplatin administered on days 1, 15, and 29. After neoadjuvant treatment, a curative gastrectomy with extended (D2) lymph node dissection was planned.

**Results:**

Nine patients were enrolled. At level 3, one patient had dose-limiting grade 3 diarrhea. Another patient experienced grade 3 nausea and intended to discontinue the treatment. Overall, because 2 of 3 patients experienced dose-limiting toxicity at level 3, we confirmed level 3 (Cisplatin 25 mg/m^2^) as the maximum tolerated dose and level 2 (Cisplatin 20 mg/m^2^) as the recommended dose (RD). The response rate was 78%, and 8 patients underwent curative gastrectomy. Resected specimens showed a histological response in 6 patients (75%), including one with a pathological complete response.

**Conclusions:**

In this phase I trial, RD of cisplatin was identified as 20 mg/m^2^. Generally, S-1 plus biweekly cisplatin can be given safely with concurrent radiation. We have initiated a multicenter phase II trial to further confirm the efficacy and safety of this approach.

**Trial registration:**

UMIN000008941

## Background

About one million new cases of stomach cancer are estimated to have occurred (988,000 cases), making it currently the fourth most common malignancy and the second leading cause of cancer deaths worldwide (736000 deaths, 9.7% of the total) [1]. At present, for resectable advanced gastric cancer, R0 resection with extended (D2) lymph node dissection has been shown to reduce gastric cancer-specific deaths [2,3]. However, in contrast to the fact that patients at an early stage show a remarkable 5-year overall survival rate of >90% [4,5], the locoregional as well as distant recurrence rate is high in advanced stages such as Stage II and III (based on the Japanese classification of gastric carcinoma) [6-8]. In order to achieve further improvements in the prognosis of advanced gastric cancer, adjuvant chemo- or chemoradiotherapy following curative gastric cancer resection is standard [9-13]. However, in highly advanced gastric cancers which have bulky primary tumors or multiple lymph node metastases, the recurrence rate remains high and the prognosis disappointing [2,6]. Therefore, there is an urgent need to establish a more intense multidisciplinary treatment for these patients.

Chemoradiotherapy is reportedly an effective intensive locoregional treatment for gastric cancer [11-14]. As an adjuvant treatment, Lee et al. showed that chemoradiotherapy led to a statistically significant prolongation of postoperative disease-free survival in gastric cancer patients with positive pathologic lymph nodes in the ARTIST trial [15]. Therefore, chemoradiotherapy could be valuable for these populations.

Neoadjuvant treatment for advanced gastric cancer patients was recently shown to be beneficial because of a higher compliance than adjuvant treatment [16-18]. Moreover, Ajani et al. showed that neoadjuvant chemoradiotherapy may prolong postoperative survival [19]. However, there was a limitation to their study as surgery with extended lymph node dissection was not performed after treatment. Therefore, we planned neoadjuvant chemoradiotherapy followed by curative gastrectomy with D2 lymph node dissection.

We previously conducted chemoradiotherapy with S-1 plus daily cisplatin and concurrent radiotherapy for unresectable or metastatic gastric cancer in the phase II study and showed its clinical benefit [20]; however, with this treatment schedule, all patients needed to be admitted during treatment. In order to deliver this treatment in an outpatient setting, S-1 plus weekly cisplatin with concurrent radiation was defined as the treatment protocol in our previous study KOGC01. Although adequate safety and efficacy were reported, myelosuppression was frequently observed, leading to treatment delay in several cases [21].

In this trial KOGC04, the treatment schedule of cisplatin was changed from weekly to biweekly because we previously showed the maintenance of the efficacy and safety of the biweekly cisplatin [22]. There was no change in the schedule of the radiation and its total dose was the same as that in the KOGC01. The aim of the present phase I study was to define the maximum tolerated dose (MTD) and dose-limiting toxicity (DLT) as well as the recommended dose (RD) for neoadjuvant chemoradiotherapy with S-1 plus biweekly cisplatin.

## Methods

### Study design

This noncomparative, dose-escalation study was conducted at Keio University Hospital, Japan, with the approval of the Ethics Committee of Keio University School of Medicine. All eligible patients provided their written informed consent to participation. The tumors were classified in accordance with the Japanese Classification of Gastric Carcinoma, 3rd English edition (JCGC) [8]. Clinical adverse events were evaluated according to the Common Terminology Criteria for Adverse Events (ver. 4.0). To evaluate treatment response, the Response Evaluation Criteria in Solid Tumors (RECIST) version 1.1 was used.

### Eligibility

All patients diagnosed with advanced gastric cancer at our institution were eligible for enrollment in this phase I study. Enrollment criteria were as follows: (1) histological diagnosis of stomach adenocarcinoma; (2) clinically measurable lymph node metastasis according to RECIST (ver. 1.1) criteria; (3) T3 or T4 tumor depth; (4) neither distant nor peritoneal metastasis identified by abdominal computed tomography (CT); (5) esophageal invasion <3 cm; (6) age between 20 and 75 years; (7) Eastern Cooperative Oncology Group (ECOG) Performance status of 0 or 1; (8) no past history of chemotherapy or chemoradiotherapy; (9) no past treatment of gastric cancer; and (10) adequate organ function (defined by white blood cell count between 3,000 and 12,000 cells/mm^3^, neutrophil count > 1,500/mm^3^, platelet count > 100,000/mm^3^, total bilirubin < 1.5 mg/dl, serum aspartate and alanine transaminase no greater than 2 times the normal upper limit, and creatinin clearance > 60 ml/min).

### Chemoradiotherapy

**Figure 1:**
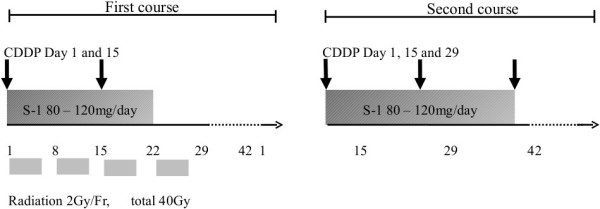
Chemoradiotherapy consisted of combination chemotherapy with S-1, biweekly cisplatin, and fractionated radiation therapy.

The chemoradiotherapy protocol consisted of administration of S-1 plus biweekly cisplatin and radiation (Figure 1). The initial chemoradiotherapy schedule was for 6 weeks: S-1 was orally administered every day on days 1–15, and the total dose was based on the patient’s body surface area (BSA), as follows: <1.25 m^2^, 80 mg; 1.25–1.5 m^2^, 100 mg; and >1.5 m^2^, 120 mg. An escalating dose of cisplatin was administered by infusion over 1 h on days 1 and 15 without infusional hydration. The starting dose (level 1) of cisplatin was 15 mg/m^2^, the second dose (level 2) was 20 mg/m^2^, and the third dose (level 3) was 25 mg/m^2^.

Radiotherapy was performed with photons from a linear accelerator with an energy **≥** 6MV, and three dimensional planning was performed. Dose constraints were as follows: both kidneys V30 ≤ 20 Gy, liver V30 ≤ 30 Gy, heart V40 ≤ 30 Gy, both lungs V20 ≤ 20 Gy, spinal cord maximum dose < 45 Gy. Clinical target volume (CTV) included the primary tumor with a 3-cm margin and metastatic lymph nodes with a 1-cm margin. Entire stomach and the perigastric and celiac lymph node stations were also delineated as part of the CTV. The planning target volume (PTV) contained the CTV with a 2-cm margin to account for setup and organ motion. To minimize interfraction variation, irradiation was performed in the early morning on an empty stomach. A total dose of 40 Gy was delivered in 2.0-Gy fractions at a rate of 5 fractions per week. The dose was prescribed to a reference point within the PTV according to the International Commission on Radiation Units and Measurements 50 and 62.

After initial chemoradiotherapy, one cycle of combination chemotherapy with S-1 plus biweekly cisplatin was delivered. This consisted of 42 days of S-1 administered from day 1 to 28 and of cisplatin administered on days 1, 15, and 29.

In order to maintain adequate renal function, all patients were recommended oral fluid management without infusional hydration during this treatment same as that in our previous report [20,22]. Regarding antiemetic medication, we routinely used 5-HT3 antagonists and dexamethasone before every cisplatin infusion. Because cisplatin was delivered fractionally, aprepitant was optional for each case.

### DLT and dose escalation method

Patients who experienced any clear treatment-related grade 4 hematological toxicity, febrile neutropenia higher than grade 3, or grade 3/4 nonhematological toxicity were considered to have experienced DLT. Other DLT events included a delay in the initiation of the 2nd cycle of chemotherapy of more than 10 days duration, a delay of more than 10 days for each cisplatin infusion, failure to complete radiation within 42 days, and surgery not performed within 42 days after the completion of the 2nd cycle of chemotherapy.

Three patients were enrolled at each dose level. If no DLT was experienced during chemoradiotherapy, an additional 3 patients were included in the next dose level. If 1 patient within the first 3 patients recruited to a level experienced DLT, another 3 patients were treated with the same dose. If more than 2 out of 6 patients experienced DLT at a dose, then that dose was defined as MTD. Furthermore, if 2 or 3 of the first 3 patients recruited to a level experienced DLT, the dose was defined as the MTD. Therefore, RD was set at one level lower than MTD.

### Clinical evaluation

Between days 29 and 42 of the 2nd cycle of chemotherapy, an upper gastrointestinal endoscopy and abdominal CT were performed for clinical evaluation based on RECIST criteria. Response to neoadjuvant treatment, resectability, and potential for surgical curability were evaluated using these assessments.

### Surgery

Patients evaluated as being able to achieve curable resection were eligible for surgery. Surgery was planned within 6 weeks after the completion of the 2nd cycle. Gastrectomy with D2 lymph node dissection was performed after we confirmed that there was no peritoneal metastasis, positive lavage cytology, or adjacent organ invasion. All resected specimens, including the lymph nodes, were examined to evaluate pathological response to the preoperative treatment according to the JGCA.

## Results

### Patient characteristics

Between 2011 and 2012, 9 patients who were diagnosed with advanced gastric cancer with lymph node metastasis were enrolled in the study. Characteristics of the 9 patients are presented in Table 1. Their median age was 62 years, and all were male with an ECOG performance status of 0. Regarding the depth of invasion of the primary tumor, in 6 patients, subserosal invasion (T3) was observed. There was also one case with invasion of the pancreas, and a gastrojejunostomy bypass was performed before enrollment. Eight of 9 patients were diagnosed with N2 lymph node metastasis, and none had suspected distant metastasis. Thus, in terms of pretreatment staging, 4 of 9 patients were diagnosed as Stage IIIA and 5 as stage IIIB.

**Table 1:** Patient and tumor characteristics

**n = 9**	**No. of patients**
Age median (range)	62 (52–77)
Sex male/female	9/0
ECOG performance status 0/1	9/0
Location U/M/L	3/2/4
Macroscopic Type 1/2/3/4	0/2/6/1
T 3(SS)/4a(SE)/4b(SI)	6/2/1
N 1/2/3	1/8/0
M 0/1	9/0
Stage IIIA/IIIB/IIIC	4/5/0

U: Upper-third of stomach; M: Middle-third of stomach; L: Lower-third of stomach; macroscopic type 1: Polypoid tumor; type 2: Ulcerated tumor with sharply demarcated and raised margins; type 3: Ulcerated tumor with infiltration; type 4: Diffusely infiltrated tumor; SS: Tumor has invaded the subserosa; SE: Tumor has penetrated the serosa; SI: Tumor has invaded adjacent structures.

### MTD and RD

Hematological and nonhematological toxicities are shown in Table 2. No hematological toxicities were greater than grade 4, which was defined as DLT. Regarding nonhematological toxicities, one patient had dose-limiting grade 3 diarrhea. In addition, because the oral intake of a patient was remarkably decreased and the patient was admitted to the hospital, we evaluated it as grade 3 anorexia. No significant weight loss was reported in this case. He intended to discontinue treatment during the 1st course. Overall, 2 of 3 patients experienced DLT at level 3 (Table 3); therefore, level 3 (Cisplatin 25 mg/m^2^) was set as MTD and level 2 (Cisplatin 20 mg/m^2^) as RD.

**Table 2:** Adverse events

		**Grade**				
		1	2	3	4	3/4
Hematological						
Leukocytopenia	Level 1 (n = 3)	1	0	0	0	0
	2 (n = 3)	1	0	1	0	1
	3 (n = 3)	1	0	0	0	0
Neutropenia	1	0	0	0	0	0
	2	0	0	1	0	1
	3	1	0	0	0	0
Anemia	1	0	0	0	0	0
	2	0	0	1	0	1
	3	0	0	0	0	0
Thrombocytopenia	1	1	0	1	0	1
	2	2	0	0	0	0
	3	0	2	0	0	0
Nonhematological						
Nausea	1	0	1	0	0	0
	2	0	0	0	0	0
	3	1	0	1*	0	1
Diarrhea	1	0	0	0	0	0
	2	0	0	0	0	0
	3	0	0	1*	0	1
AST/ALT elevation	1	1	0	0	0	0
	2	2	0	0	0	0
	3	1	0	0	0	0
Bilirubin elevation	1	1	1	0	0	0
	2	0	1	0	0	0
	3	1	0	0	0	0
Creatinin elevation	1	1	0	0	0	0
	2	0	0	0	0	0
	3	1	0	0	0	0

*: Dose-limiting toxicity, AST: Aspartate aminotransferase, ALT: Alanine aminotransferase.

**Table 3:** Dose-limiting toxicity

**n = 9**	**Level 1**	Level 2**	Level 3*
Hematological toxicity	0	0	0
Nonhematological toxicity	0	0	2
Treatment delay	0	0	0

*: Maximum tolerated dose, **: Recommended dose.

### Clinical Efficacy

Patient outcomes are shown in Table 4. Overall, 7 of 9 patients achieved a clinical partial response (PR), resulting in a response rate of 78%. After neoadjuvant therapy, 8 of 9 patients underwent a gastrectomy with D2 (extended) lymph node dissection. One patient who experienced DLT intended to discontinue treatment during the 1st course. The median operating time was 249 min, and the median blood loss was 625 ml. Regarding postoperative complications, one patient encountered stump leakage due to a pancreatic fistula, and another patient developed chylous ascites. Both were treated with drainage alone, and there were no treatment-related deaths. Pathological examination of resected specimens showed that the therapeutic efficacy was histologically classified as grade 1a in two patients, grade 1b in one patient, grade 2 in 4 patients, and grade 3 in one patient. Six of 8 (75%) patients who underwent surgery showed a histological response to chemoradiotherapy, including one patient with a pathological complete response.

**Table 4:** Patient outcomes

**n = 9**	**No. of patients**
Clinical Response	
PD/SD/PR/CR (Response rate)	0/2/7/0 (78%)
Level 1	0/1/2/0
Level 2	0/0/3/0
Level 3	0/1/2/0
Surgery	
Distal/Total	4/4
D1/D2	0/8
R0/R1/R2	8/0/0
Operation time (min)	249 (195–288)
Blood loss (ml)	578 (110–1700)
Postoperative complication	
Stump leakage	1
Pancreatic fistula	1
Chylous ascites	1
Histological therapeutic effect	
Grade 1a/1b/2/3	2/1/4/1

PD: Progressive disease; SD: Stable disease; PR: Partial response; CR: Complete response; Distal: Distal gastrectomy; Total: Total gastrectomy; D1: Limited lymphadenectomy; D2: Extended lymphadenectomy; R0: No residual tumor; R1: Microscopic residual tumor; R2: Macroscopic residual tumor.

## Discussion

Intensive multidisciplinary treatment is currently required for highly advanced gastric cancer, and chemoradiotherapy is a potent option. In the ARTIST trial, which compared adjuvant chemoradiotherapy and chemotherapy alone, a significant reduction in postoperative recurrence was reported in patients who had histologically proven metastatic lymph nodes [15]. Thus, in this study, efficacy was achieved among patients with strongly suspected lymph node metastasis preoperatively.

In contrast to the fact that capecitabin was combined with cisplatin in the ARTIST study, the S-1 was included in our treatment. The S-1 plus cisplatin treatment schedule is one of the current standards of care in Japan for patients with unresectable or recurrent gastric cancer [23]. Using chemoradiotherapy including S-1, we previously completed a phase II trial for patients with unresectable or metastatic gastric cancer and showed the tolerability and efficacy of this treatment schedule [20]. In the ARTIST study, the total cisplatin dose was 60 mg/m^2^. In this phase I study, although cisplatin was fractionally delivered, its total dose was the same as that in the ARTIST study.

In general, intensive treatments such as chemoradiotherapy are believed to weaken the general condition of patients and increase postoperative complications. In fact, in our previous study KOGC01, several patients could not follow the treatment protocol because of hematological toxicities. In this study, we changed the chemotherapy schedule for infusional cisplatin from weekly to biweekly regimen, which was conducted for unresectable gastric cancer patients and found to be safe [22]. Consequently, the incidence of grade 3 or 4 hematological toxicity decreased from 40% to 22%, and 8 out of 9 participants could complete the neoadjuvant chemoradiotherapy regimen. Major toxicities encountered in the present study were nonhematological toxicities, including nausea and diarrhea, and these could be managed and were tolerable. Although two patients experienced postoperative complications, including chylous ascites and a pancreatic fistula followed by duodenal stump leakage, they were treated conservatively, which shows that our regimen is tolerable. Overall, level 3 (Cisplatin 25 mg/m^2^) was set as MTD and level 2 (Cisplatin 20 mg/m^2^) as RD.

We previously confirmed a marked response to chemoradiotherapy and an improved prognosis among patients with unresectable or metastatic gastric cancer [20]. Because curative gastrectomy for highly advanced gastric cancer is invasive, achieving tumor reduction before surgery can reduce perioperative risks. Thus, our protocol, which had a high response rate, is favorable as a neoadjuvant treatment. Although our ability to evaluate efficacy was limited in this phase I trial, we did achieve a histological response rate of 75%, including one case with a pathological complete response. Therefore, the exact treatment efficacy was maintained after the change of cisplatin schedule from weekly to biweekly to reduce the toxicities.

As previously reported, chemoradiotherapy was effective especially for locally advanced gastric cancer [4,5,15-21]. Particularly, neoadjuvant chemotherapy is considered to have several clinical benefits such as a reduction in the viability of micrometastases, an increase in the rate of curability, an enhancement in treatment compliance, and an improvement in the evaluation of chemosensitivity. Furthermore, the addition of radiation could improve local control. Therefore, we think that there were several clinical benefits of neoadjuvant CRT in patients with advanced gastric cancer, especially in cases with bulky primary tumor or multiple lymph node metastases and those patients are preferred participants. In contrast, patients with scirrhous-type gastric cancer, which tends to disseminate to the peritoneum, may not find chemoradiotherapy beneficial.

Nonetheless the recent advancement of technology, radiotherapy for gastric cancer is still challenging. Precise target and organ delineation and dose-volume calculation is evolving with three-dimensional conformal radiotherapy. However, uncertainties arising from variations in stomach filling and respiratory motion still remain [24]. To resolve those issues, in the current study, appropriate margins were prepared and irradiation was performed on an empty stomach. Margins must be modified to maintain dose constraint for the organ at risk. As a result, we found no enlarged lymph node, which interfered gastrectomy with extended lymph node dissection during operation. Notably, surgical complication considered to possibly be induced by influence of radiation was not increased. Further advanced treatment technique such as intensity-modulated radiation therapy and/or image-guided radiation therapy may allow more excellent target coverage and normal structure sparing, and led to superior treatment outcomes [25].

## Conclusions

In this phase I study, RD of cisplatin was established as 20 mg/m^2^, and the efficacy and safety of S-1 plus cisplatin with concurrent radiation was confirmed for patients with advanced gastric cancer and lymph node metastasis. In order to provide further confirmation of the efficacy and safety of this approach, we have initiated a multicenter phase II trial.

## Competing interests

The authors declare that they have no competing interests.

## Authors’ contributions

SM and JF contributed to data analysis and drafted the manuscript; TT provided a critical review of the manuscript; KF was responsible for the unified management of patients’ data; TT and HK conducted gastrectomy with extended lymph node dissection in this study; OK and NS managed the protocol of radiation therapy; YS, HT and YK were responsible for the conception of the study and provided the final approval of the version for publication. And all authors have read and approved the final manuscript.

## References

[B1] Ferlay J Shin HR Bray F Forman D Mathers C Parkin DM Estimates of worldwide burden of cancer in 2008: GLOBOCAN 2008. Int J Cancer 2010 127 2893 917 10.1002/ijc.25516 21351269

[B2] Songun I Putter H Kranenbarg EM Sasako M van de Velde CJ Surgical treatment of gastric cancer: 15-year follow-up results of the randomised nationwide Dutch D1D2 trial Lancet Oncol 2010 11 439 49 10.1016/S1470-2045(10)70070-X 20409751

[B3] Wu CW Hsiung CA Lo SS Hsieh MC Chen JH Li AF Lui WY Whang-Peng J Nodal dissection for patients with gastric cancer: a randomised controlled trial Lancet Oncol 2006 7 309 15 10.1016/S1470-2045(06)70623-4 16574546

[B4] Maruyama K Kaminishi M Hayashi K Isobe Y Honda I Katai H Arai K Kodera Y Nashimoto A Japanese Gastric Cancer Association Registration Committee Gastric cancer treated in 1991 in Japan: data analysis of nationwide registry Gastric Cancer 2006 9 51 66 16767357 10.1007/s10120-006-0370-y

[B5] Sano T Sasako M Kinoshita T Maruyama K Recurrence of early gastric cancer. Follow-up of 1475 patients and review of the Japanese literature Cancer 1993 72 3174 8 10.1002/1097-0142(19931201)72:11<3174::AID-CNCR2820721107>3.0.CO;2-H 8242540

[B6] Sasako M Sakuramoto S Katai H Kinoshita T Furukawa H Yamaguchi T Nashimoto A Fujii M Nakajima T Ohashi Y Five-year outcomes of a randomized phase III trial comparing adjuvant chemotherapy with S-1 versus surgery alone in stage II or III gastric cancer J Clinical Oncol 2011 29 4387 93 10.1200/JCO.2011.36.5908 22010012

[B7] Yoo CH Noh SH Shin DW Choi SH Min JS Recurrence following curative resection for gastric carcinoma Br J Surg 2000 87 236 42 10.1046/j.1365-2168.2000.01360.x 10671934

[B8] Japanese Gastric Cancer Association Japanese classification of gastric carcinoma: 3rd English edition Gastric Cancer 2011 14 101 12 21573743 10.1007/s10120-011-0041-5

[B9] Sakuramoto S Sasako M Yamaguchi T Kinoshita T Fujii M Nashimoto A Furukawa H Nakajima T Ohashi Y Imamura H Higashino M Yamamura Y Kurita A Arai K ACTS-GC Group Adjuvant chemotherapy for gastric cancer with S-1, an oral fluoropyrimidine New Engl J Med 2007 357 1810 20 10.1056/NEJMoa072252 17978289

[B10] Cunningham D Allum WH Stenning SP Thompson JN Van de Velde CJ Nicolson M Scarffe JH Lofts FJ Falk SJ Iveson TJ Smith DB Langley RE Verma M Weeden S Chua YJ MAGIC Trial Participants Perioperative chemotherapy versus surgery alone for resectable gastroesophageal cancer New Engl J Med 2006 355 11 20 10.1056/NEJMoa055531 16822992

[B11] Macdonald JS Smalley SR Benedetti J Hundahl SA Estes NC Stemmermann GN Haller DG Ajani JA Gunderson LL Jessup JM Martenson JA Chemoradiotherapy after surgery compared with surgery alone for adenocarcinoma of the stomach or gastroesophageal junction New Engl J Med 2001 345 725 30 10.1056/NEJMoa010187 11547741

[B12] Smalley SR Benedetti JK Haller DG Hundahl SA Estes NC Ajani JA Gunderson LL Goldman B Martenson JA Jessup JM Stemmermann GN Blanke CD Macdonald JS Updated analysis of SWOG-directed intergroup study 0116: a phase III trial of adjuvant radiochemotherapy versus observation after curative gastric cancer resection J Clin Oncol 2012 30 2327 33 10.1200/JCO.2011.36.7136 22585691 PMC4517071

[B13] Japanese Gastric Cancer Association Japanese gastric cancer treatment guidelines 2010 (ver. 3) Gastric Cancer 2011 14 113 123 21573742 10.1007/s10120-011-0042-4

[B14] Kassam Z Mackay H Buckley CA Fung S Pintile M Oza A Brierley J Swallow C Cummings B Knox JJ Kim J Wong R Siu L Feld R Ringash J Adjuvant chemoradiation for gastric cancer with infusional 5-fluorouracil and cisplatin: a phase I study Curr Oncol 2010 17 34 41 20697512 10.3747/co.v17i4.521 PMC2913826

[B15] Lee J Lim do H Kim S Park SH Park JO Park YS Lim HY Choi MG Sohn TS Noh JH Bae JM Ahn YC Sohn I Jung SH Park CK Kim KM Kang WK Phase III trial comparing capecitabine plus cisplatin versus capecitabine plus cisplatin with concurrent capecitabine radiotherapy in completely resected gastric cancer with D2 lymph node dissection: the ARTIST trial J Clin Oncol 2012 30 268 273 10.1200/JCO.2011.39.1953 22184384

[B16] Yoshikawa T Sasako M Yamamoto S Sano T Imamura H Fujitani K Oshita H Ito S Kawashima Y Fukushima N Phase II study of neoadjuvant chemotherapy and extended surgery for locally advanced gastric cancer Br J Surg 2009 96 1015 22 10.1002/bjs.6665 19644974

[B17] Nashimoto A Yabusaki H Nakagawa S Takii Y Tsuchiya Y Otsuo T Preoperative chemotherapy with S-1 and cisplatin for highly advanced gastric cancer Anticancer Res 2009 29 4689 96 20032421

[B18] Inoue T Yachida S Usuki H Kimura T Hagiike M Okano K Suzuki Y Pilot feasibility study of neoadjuvant chemoradiotherapy with S-1 in patients with locally advanced gastric cancer featuring adjacent tissue invasion or JGCA bulky N2 lymph node metastases Ann Surg Oncol 2012 19 2937 45 10.1245/s10434-012-2332-4 22466666

[B19] Ajani JA Mansfield PF Janjan N Morris J Pisters PW Lynch PM Feig B Myerson R Nivers R Cohen DS Gunderson LL Multi-institutional trial of preoperative chemoradiotherapy in patients with potentially resectable gastric carcinoma J Clin Oncol 2004 22 2774 80 10.1200/JCO.2004.01.015 15254045

[B20] Saikawa Y Kubota T Kumagai K Nakamura R Kumai K Shigematsu N Kubo A Kitajima M Kitagawa Y Phase II study of chemoradiotherapy with S-1 and low-dose cisplatin for inoperable advanced gastric cancer Int J Radiat Oncol Biol Phys 2008 71 173 9 10.1016/j.ijrobp.2007.09.010 17996385

[B21] Takahashi T Saikawa Y Takaishi H Takeuchi H Wada N Oyama T Fukuda K Fukada J Kawaguchi O Shigematsu N Kitagawa Y Phase I study of neoadjuvant chemoradiotherapy consisting of S-1 and cisplatin for patients with resectable advanced gastric cancer (KOGC-01) Anticancer Res 2011 31 3079 83 21868563

[B22] Takahashi T Saikawa Y Takaishi H Takeuchi H Wada N Oyama T Nakamura R Kitagawa Y Feasibility and efficacy of combination chemotherapy with S-1 and fractional Cisplatin for advanced gastric cancer Anticancer Res 2010 30 3759 62 20944165

[B23] Koizumi Y Nakahara H Hara T Takagane A Akiya T Takagi M Miyashita K Nishizaki T Kobayashi O Takitama W Toh Y Nagaie T Takagi S Yamamura Y Yanaoka K Orita H Takeuchi M S-1 plus cisplatin versus S-1 alone for first-line treatment of advanced gastric cancer (SPIRITS trial): a phase III trial Lancet Oncol 2008 9 215 221 10.1016/S1470-2045(08)70035-4 18282805

[B24] Wysocka B Kassam Z Lockwood G Brierley J Dawson LA Buckley CA Jaffray D Cummings B Kim J Wong R Ringash J Interfraction and respiratory organ motion during conformal radiotherapy in gastric cancer Int J Rad Oncol Bio Phys 2010 77 53 59 10.1016/j.ijrobp.2009.04.046 19665320

[B25] Chakravarty T Crane CH Ajani JA Mansfield PF Briere TM Beddar AS Mok H Reed VK Krishnan S Delclos ME Das P Intensity-modulated radiation therapy with concurrent chemotherapy as preoperative treatment for localized gastric adenocarcinoma Int J Rad Oncol Bio Phys 2012 83 581 586 10.1016/j.ijrobp.2011.07.035 22137021

