# Correction: phase I study of neoadjuvant chemoradiotherapy with S-1 plus biweekly cisplatin for advanced gastric cancer patients with lymph node metastasis -KOGC04-

**DOI:** 10.1186/1748-717X-9-140

**Published:** 2014-06-25

**Authors:** Satoru Matsuda, Tsunehiro Takahashi, Junichi Fukada, Kazumasa Fukuda, Hirofumi Kawakubo, Yoshiro Saikawa, Osamu Kawaguchi, Hiroya Takeuchi, Naoyuki Shigematsu, Yuko Kitagawa

**Affiliations:** 1 Department of Surgery, Keio University School of Medicine, 35 Shinanomachi, Shinjuku-ku, Tokyo 160-8582, Japan; 2 Department of Radiology, Keio University School of Medicine, 35 Shinanomachi, Shinjuku-ku, Tokyo 160-8582, Japan

## Correction

After the publication of this work [1], we noted that owing to an inadvertent mistake, the description of the chemotherapy schedule shown in the Methods was different from that shown in the Abstract and the duration of S-1 treatment in the initial chemoradiotherapy was inadequately described in Figure 1. Therefore, Methods and Figure 1 were modified accordingly.

**Figure 1:**
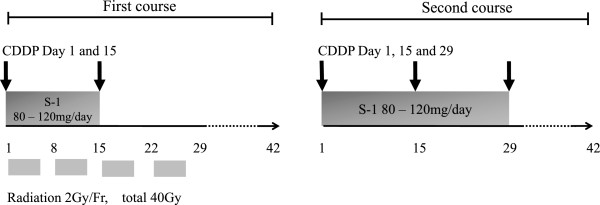
Chemoradiotherapy consisted of combination chemotherapy with S-1, biweekly cisplatin, and fractionated radiation therapy.

In the “Chemoradiotherapy” section in the Methods, the 1st and 3rd paragraphs should read as follows:

“The chemoradiotherapy protocol consisted of administration of S-1 plus biweekly cisplatin and radiation (Figure 1). The initial chemoradiotherapy schedule was for 6 weeks: S-1 was orally administered twice daily from the evening of day 1 to the morning of day 15, and the total dose was based on the patient’s body surface area (BSA), as follows: <1.25 m^2^, 80 mg; 1.25–1.5 m^2^, 100 mg; and >1.5 m^2^, 120 mg. An escalating dose of cisplatin was administered by infusion over 1 h on days 1 and 15 without infusional hydration. The starting dose (level 1) of cisplatin was 15 mg/m^2^, the second dose (level 2) was 20 mg/m^2^, and the third dose (level 3) was 25 mg/m^2^.”

“After initial chemoradiotherapy, one cycle of combination chemotherapy with S-1 plus biweekly cisplatin was delivered. This consisted of 42 days of S-1 administered from the evening of day 1 to the morning of day 29 and of cisplatin administered on days 1, 15, and 29.”In “Figure 1,” based on the description in the manuscript, the duration of S-1 treatment in the initial chemoradiotherapy was revised to 1–15 days from 1–22 days (Figure 1).
